# Evaluating an automated machine learning model that predicts visual acuity outcomes in patients with neovascular age-related macular degeneration

**DOI:** 10.1007/s00417-021-05544-y

**Published:** 2022-02-05

**Authors:** Abdallah Abbas, Ciara O’Byrne, Dun Jack Fu, Gabriella Moraes, Konstantinos Balaskas, Robbert Struyven, Sara Beqiri, Siegfried K. Wagner, Edward Korot, Pearse A. Keane

**Affiliations:** 1 University College London Medical School, London, UK; 2 Moorfields Eye Hospital, London, UK; 3 School of Medicine, Trinity College, Dublin, Ireland; 4 NIHR Biomedical Research Centre at Moorfields Eye Hospital, UCL Institute of Ophthalmology, London, UK; 5 Stanford University Byers Eye Institute, Palo Alto, CA USA

**Keywords:** Automated machine learning, Neovascular age-related macular degeneration, Artificial intelligence, Anti-VEGF, OCT, Model interpretability

## Abstract

**Purpose:**

Neovascular age-related macular degeneration (nAMD) is a major global cause of blindness. Whilst anti-vascular endothelial growth factor (anti-VEGF) treatment is effective, response varies considerably between individuals. Thus, patients face substantial uncertainty regarding their future ability to perform daily tasks. In this study, we evaluate the performance of an automated machine learning (AutoML) model which predicts visual acuity (VA) outcomes in patients receiving treatment for nAMD, in comparison to a manually coded model built using the same dataset. Furthermore, we evaluate model performance across ethnic groups and analyse how the models reach their predictions.

**Methods:**

Binary classification models were trained to predict whether patients’ VA would be ‘Above’ or ‘Below’ a score of 70 one year after initiating treatment, measured using the Early Treatment Diabetic Retinopathy Study (ETDRS) chart. The AutoML model was built using the Google Cloud Platform, whilst the bespoke model was trained using an XGBoost framework. Models were compared and analysed using the What-if Tool (WIT), a novel model-agnostic interpretability tool.

**Results:**

Our study included 1631 eyes from patients attending Moorfields Eye Hospital. The AutoML model (area under the curve [AUC], 0.849) achieved a highly similar performance to the XGBoost model (AUC, 0.847). Using the WIT, we found that the models over-predicted negative outcomes in Asian patients and performed worse in those with an ethnic category of Other. Baseline VA, age and ethnicity were the most important determinants of model predictions. Partial dependence plot analysis revealed a sigmoidal relationship between baseline VA and the probability of an outcome of ‘Above’.

**Conclusion:**

We have described and validated an AutoML-WIT pipeline which enables clinicians with minimal coding skills to match the performance of a state-of-the-art algorithm and obtain explainable predictions.

**Supplementary Information:**

The online version contains supplementary material available at 10.1007/s00417-021-05544-y.

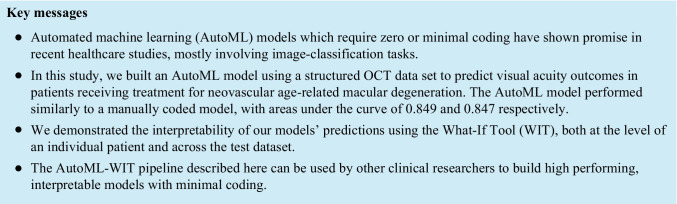



## Introduction

Age-related macular degeneration (AMD) affects an estimated 200 million people worldwide and is the most common cause of blindness in the developed world [1]. Up to 90% of cases involving blindness are attributed to neovascular AMD (nAMD), where new growth of structurally fragile blood vessels causes fluid to leak and damage the macula [2]. This leads to rapid loss of central vision that resulting in physical disability and significant psychological stress, with many patients fearing loss of independence [3]. Whilst anti-vascular endothelial growth factor (anti-VEGF) injections are an effective treatment [4, 5], response varies considerably between patients, and real-world treatment outcomes often do not match clinical trials [6]. Therefore, the ability to accurately predict how an individual’s visual acuity (VA) will change in response to treatment may be highly desirable.

A significant amount of real-world structured data has now been collected from patients receiving anti-VEGF treatment for nAMD. This includes VA scores at each appointment and measurements taken from optical coherence tomography (OCT) scans, a non-invasive imaging modality routinely used to determine treatment indication and therapeutic response in AMD. In 2018, Rohm et al. used this data to build a machine learning (ML) model that predicts VA 12 months into the future [7]. Notably, this relied on input data collected after an initial loading of three anti-VEGF injections, rather than at baseline (i.e., immediately prior to initiation of treatment). Consequently, this model is unable to alleviate the uncertainty and anxiety that patients face when starting treatment [8]. More recently, a classification algorithm which predicts VA outcomes after a year of treatment achieved an area under the receiver operating characteristic curve (AUROC) of 0.78 [9]. Whilst this study did use baseline data, the focus was on assessing the predictive utility of quantitative OCT biomarkers. As such, demographic information was omitted despite age being a known predictor of VA outcomes [10].

Thus far, these attempts have adopted conventional ML methods. This involves investing significant time and skill into model architecture selection, data pre-processing and hyperparameter tuning. In contrast, automated machine learning (AutoML) techniques seek to accomplish these steps without user input. Recent studies assessing the feasibility of AutoML in healthcare have found promising results in comparison to bespoke models [11–14]. This represents an opportunity to enable clinicians with no computational background to leverage the power of ML.

In this retrospective cohort study, we aim to evaluate whether an AutoML model, built using the Google Cloud AutoML Tables platform, can predict VA outcomes in patients with nAMD. Specifically, we use baseline data to predict whether patients’ vision will be above or below the legal driving standard after one year of anti-VEGF treatment. Current research into AutoML has focused on image classification tasks, with few studies analysing how the technology performs relative to conventional ML when using structured data. Therefore, we evaluate the performance of our AutoML model against a bespoke model, designed in the traditional manner by computer scientists, that was trained and tuned using the same dataset.

Whilst AutoML may help to democratise artificial intelligence (AI), lack of interpretability into how models reach their decisions still represents a barrier to extensive model interrogation and buy-in [15]. Examples of algorithms which harbour entrenched racial biases have also been reported [16, 17]. Therefore, we further aim to evaluate the performance of our models across ethnic groups and analyse how they arrive at their decisions, both at the level of the individual patient and more broadly. To this end, we utilise the What-if Tool (WIT), a novel open-source AI interpretability tool [18].

## Methods

**Fig. 1:**
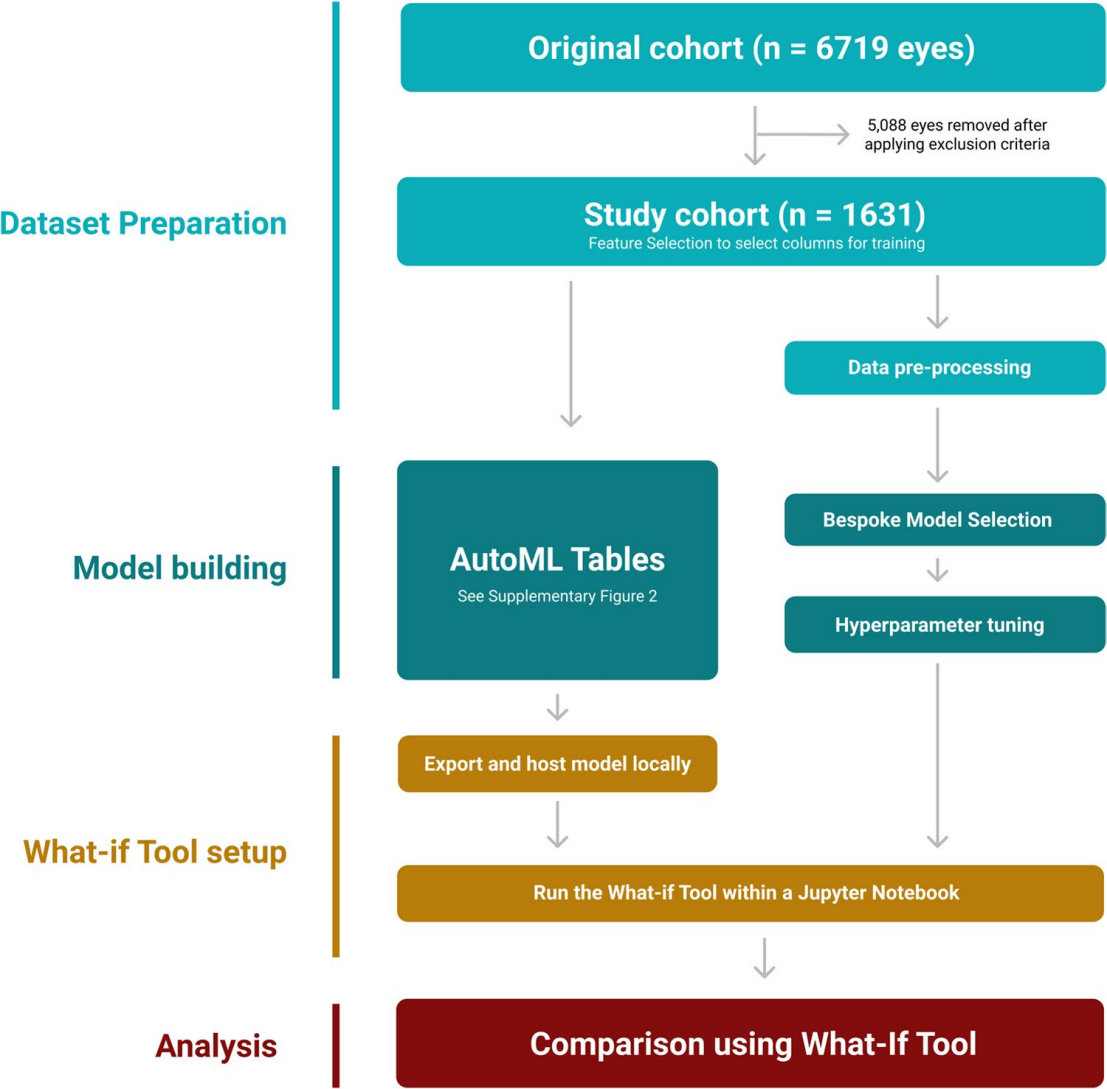
Summary of project workflow

The overall project workflow is described in Fig. 1. All programming (see the ‘Code availability’ section) was carried out using Python 3.7.9. We refer to this code in the text as Script 1 and Script 2.

### Study cohort

This study included data from patients receiving anti-VEGF (aflibercept or ranibizumab) treatment for nAMD at Moorfields Eye Hospital (MEH) between June 2012 and June 2017. Patients at MEH receive a standardised treatment consisting of an initial loading phase of three injections, four weeks apart, followed by a treat-and-extend (T&E) regimen. Although this approach is taken in the vast majority of patients, there is flexibility for individual clinicians to personalise this treatment, for example by adopting a pro re nata (PRN) approach.

Details of 169,703 appointments attended by 3392 patients were collated from MEH electronic health records. Patients were excluded if they had missing VA measurements at baseline or one year, no OCT scan at baseline or if they had previously received treatment for nAMD (Supplementary Fig. 1). After applying exclusion criteria, our final study cohort comprised of 1631 eyes from 1547 patients.

### Dataset preparation

For each eye, we recorded baseline VA, OCT-derived volume measurements and the patient’s age, gender and ethnicity.

Baseline VA was measured using the method described in the landmark Early Treatment Diabetic Retinopathy Study (ETDRS) [19]. The maximum VA score using this method is 100 (equivalent to a Snellen test score of 20/10), the requirement for recreational driving in the UK is 70 (20/40) and a score of 35 (20/200) or below is classified as legal blindness.

**Fig.2:**
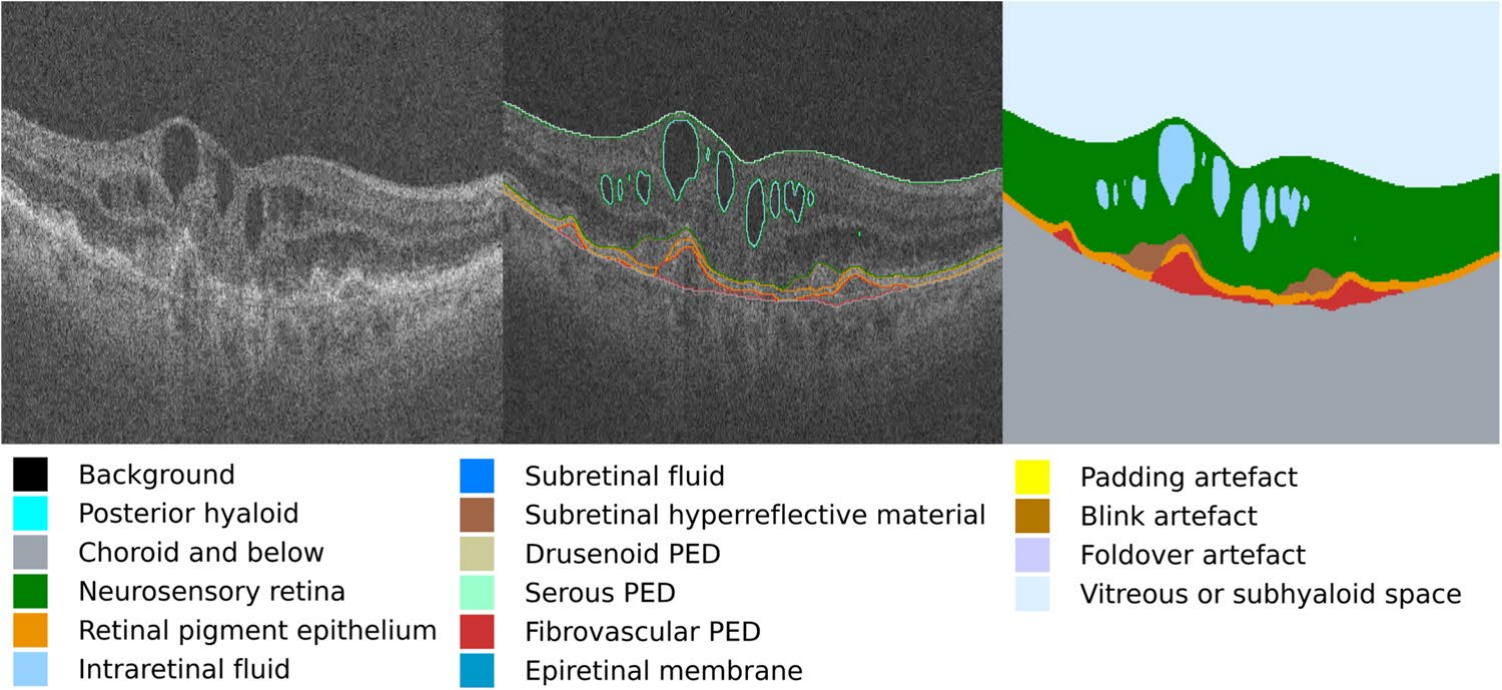
Segmentation of retinal compartments using deep learning algorithm. Exemplar OCT scan and segmentation map for a patient with neovascular age-related macular degeneration. The colour key shows the features quantified by the segmentation algorithm. Volumes outputted were scaled from voxels (2.60 × 11.72 × 47.24 $\mu m$ cuboids) to cubic millimetres before their use as input features in this study. PED = pigment epithelium detachment

For each eye, volume measurements of retinal compartments were extracted from baseline OCT scans by a deep learning segmentation algorithm (Fig. 2), as previously described [20].

Previous literature has identified the OCT-derived measurements that are important in determining VA outcomes in AMD patients [21–24]. Based on these studies, we selected the following features from those provided by the segmentation model: intraretinal fluid (IRF), subretinal fluid (SRF), subretinal hyperreflective material (SHRM), hyperreflective foci (HRF), retinal pigment epithelium (RPE) and pigment epithelium detachment (PED). PED was calculated by summing fibrovascular PED, drusenoid PED and serous PED. Feature descriptions are available in Supplementary Table 1.

To create the outcome labels for our binary classification task, we utilised patients’ VA scores at 12 months and assigned a label of ‘Above’ to patients with a VA ≥ 70, and a label of ‘Below’ for the remaining patients with VA < 70. Whilst this threshold score of 70 corresponds to the legal standard for driving, our aim was not to develop an algorithm that predicts who will be capable of driving in 1 year (since for recreational driving it is sufficient to meet the standard using both eyes) but to use a threshold that intuitively relates to a daily task and is consistent with previous literature [9, 25].

Following feature selection and labelling, we carried out a random 85:15 stratified train-test split (Supplementary Table 2). The test data was held back for evaluation using the WIT, whilst the remaining data was used for model training and validation.

AutoML Tables is an automated machine learning tool available on the Google Cloud Platform (GCP) which accepts structured data as input and automatically trains predictive models [26]. Whilst alternative platforms exist, this study focuses on AutoML Tables due to its free trial option, built-in interpretability features and higher reported performance when benchmarked against other platforms in Kaggle competitions [27]. We have created a diagram summarising our AutoML Tables workflow, as well as the steps automated by the platform (Supplementary Fig. 2). Final hyperparameter settings for our trained model are available in Supplementary Table 3.

### Bespoke model

We explored various potential algorithms including logistic regression, K-nearest neighbours, EXtreme Gradient Boosting (XGBoost) and Keras Deep Neural Networks. Initial evaluation of these options on our training and validation data yielded XGBoost as the optimal algorithm for our bespoke model.

XGBoost is considered a state-of-the-art algorithm for classification tasks using structured data [28] and is frequently used in healthcare research for predictive modelling [29–31]. XGBoost works by building an ensemble of decision trees, each of which has a set of criteria that it ‘judges’ the input data by. For example, the first decision tree may include a criterion of ‘Age > 67’. A score is then assigned based on whether the data fits this criterion or not. Evidently, making a prediction based on a single criterion is a crude method, and so in model training, subsequent trees learn their own criteria which are finetuned to correct the residual error of the prior ensemble [32].

Following algorithm selection, we carried out a grid search of 6318 different hyperparameter combinations using stratified cross-validation with ten splits (Script 2). This yielded the final hyperparameter settings used to train our model (Supplementary Table 4), ready to be evaluated on the un-seen test set.

### Feature importance

The AutoML Tables platform returns feature importance based on Shapley values, which it approximates using the sampled Shapley method [33]. Shapley values are commonly utilised in cooperative game theory and are adopted in ML to characterise the average marginal contribution of each feature to the model’s overall prediction. This provides a robust route to evaluating which features are most important for the model’s decisions in a manner consistent with human intuition [34]. We also calculated feature importance for our XGBoost model (Script 3). This was implemented using the TreeExplainer method from the SHapley Additive exPlanations (SHAP) Python package [35]. Feature importance values were normalised to sum to 1 for each model.

### What-if Tool

The WIT is an open-source, model-agnostic AI interpretability tool available to use as a Jupyter notebook extension [18]. It enables performance metrics to be analysed across different patient subgroups, giving insight into model fairness and bias. Furthermore, it allows the user to view how model predictions change when input features are hypothetically varied. This may be at the level of an individual patient or across the entire test set.

To the best of our knowledge, we are the first to analyse an AutoML model using the WIT (Script 4). Whilst the graphical user interface is intuitive to use, the initial set-up process requires some Python programming and use of the command-line interface. We have created a video (see Supplementary Information) to outline the process and enable those from a non-computational background to reproduce this method.

## Results

### Study cohort

Our cohort (summarised in Table 1) consisted of 1631 eyes from 1547 patients. The median age was 80 (IQR 73–85) with a baseline VA of 58 (IQR 46–68). Females accounted for 60% of the cohort. Ethnicities were 53% White, 11% Asian, 2% Black, 23% Other and 11% Unknown. These figures are in line with the epidemiology of AMD [36]. Eyes with an outcome label of ‘Above’ were on average younger (78 vs. 81 years, *p* < 0.01) with a higher baseline VA (67 vs. 50, *p* < 0.01) and lower volumes of IRF, SHRM and PED at baseline (all with *p* < 0.01).

**Table 1:** Input feature summary statistics categorised by outcome label. For continuous variables, we report the median (Q1–Q3); as using the Shapiro–Wilk test, all were found to have non-normal distributions. Differences between outcome groups were analysed using the Mann–Whitney *U* test for continuous variables and Fisher’s exact test for categorical variables. *HRF to 5.d.p: yotal = 0.00071 (0.00021–0.00236); Above = 0.00061 (0.00016–0.00211) and Below = 0.00077 (0.00023–0.00256)

		Total (*n* = 1631)	Above (*n* = 663)	Below (*n* = 968)	*p*-value
Age (years)		80 (73–85)	78 (71–83)	81 (75–86)	< 0.01
Ethnicity	White	868 (53.2%)	388 (58.5%)	480 (49.6%)	< 0.01
	Asian	170 (10.4%)	64 (9.7%)	106 (11.0%)	0.41
	Black	33 (2.0%)	18 (2.7%)	15 (1.5%)	0.11
	Other	380 (23.3%)	131 (19.8%)	249 (25.7%)	0.01
	Unknown	180 (11.0%)	62 (9.4%)	118 (12.2%)	0.08
Gender	Female	988 (60.6%)	400 (60.3%)	588 (60.7%)	0.88
	Male	643 (39.4%)	263 (39.7%)	380 (39.3%)	0.88
Baseline VA (ETDRS)		58 (46–68)	67 (60–70)	50 (38–60)	< 0.01
OCT Features (mm^3^)	RPE	0.81 (0.77–0.86)	0.83 (0.78–0.87)	0.80 (0.76–0.85)	< 0.01
	IRF	0.00 (0.00–0.08)	0.00 (0.00–0.03)	0.01 (0.00–0.13)	< 0.01
	SRF	0.20 (0.03–0.57)	0.19 (0.03–0.60)	0.20 (0.03–0.55)	0.85
	HRF*	0.00 (0.00–0.00)	0.00 (0.00–0.00)	0.00 (0.00–0.00)	0.01
	SHRM	0.13 (0.02–0.41)	0.07 (0.01–0.25)	0.19 (0.04–0.55)	< 0.01
	PED	0.37 (0.13–0.90)	0.28 (0.10–0.71)	0.44 (0.17–1.04)	< 0.01

### Overall model performance

**Fig.3:**
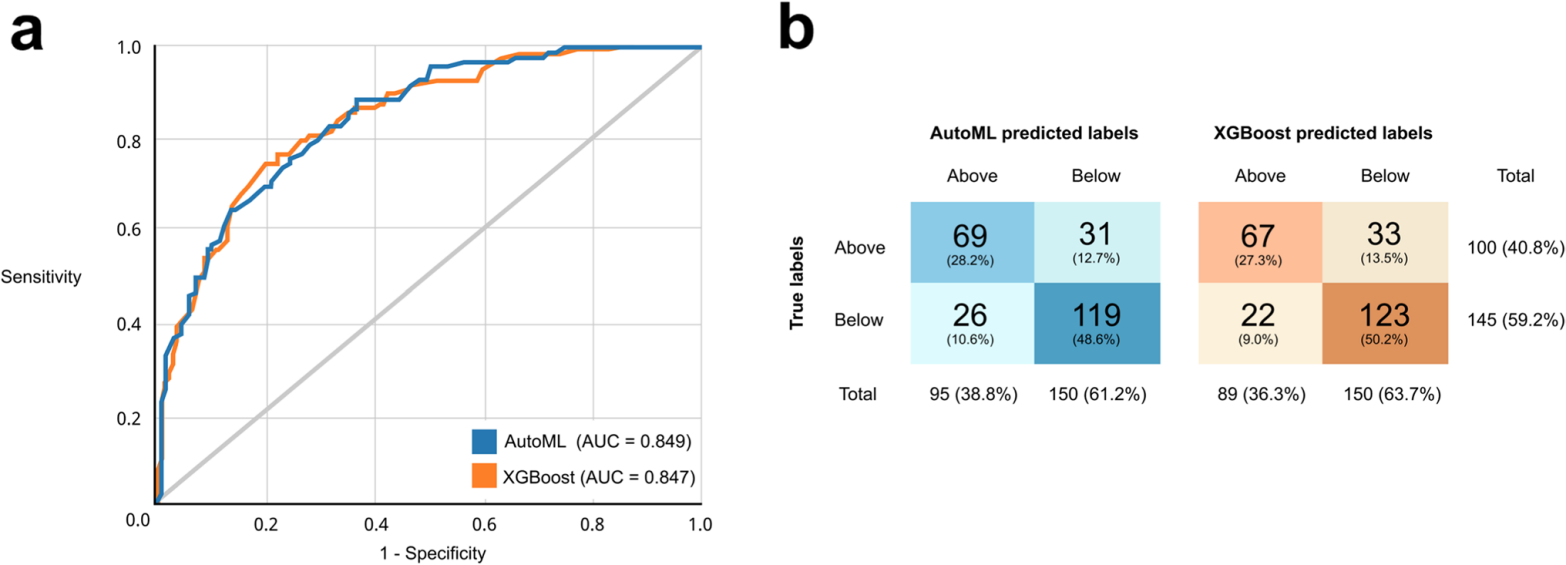
Receiver operating characteristic (ROC) curves and confusion matrices for AutoML and XGBoost models. **a** ROC curves for both models on test data, showing discriminative performance at predicting whether patients with nAMD would have a VA ‘Above’ or ‘Below’ 70 after one year of treatment. Grey line represents a random classifier. **b** Confusion matrices for AutoML and XGBoost models. Predicted labels were assigned using the default classification threshold of 0.5

The test set AUROC was 0.849 for the AutoML Tables model and 0.847 for the XGBoost model (Fig. 3a). Using DeLong’s test (Script 5), these AUROC were not found to be significantly different (*p* = 0.71). We also report the confusion matrices for both models (Fig. 3b) and further performance metrics (Table 2), highlighting the similarity between the two models.

**Table 2:** Summary performance metrics for the AutoML Tables and XGBoost models. Metrics were calculated at the default classification threshold of 0.5. *PPV* positive predictive value. *NPV* negative predictive value

	AUROC	Sensitivity	Specificity	PPV	NPV	Accuracy	F1 score
AutoML	0.849	69.0%	82.1%	72.6%	79.3%	76.7%	0.71
XGBoost	0.847	67.0%	84.8%	75.3%	78.8%	77.6%	0.71

### Analysing model performance by ethnic group

Using the WIT, we found that the AutoML model had a sensitivity of just 56.3% in Asian patients and was over-predicting negative outcomes. This issue manifested at the default classification threshold of 0.5, meaning that the model must reach a predicted probability of at least 50% to classify an eye as ‘Above’. Using the WIT to reduce the classification threshold to 0.4 helped rectify the model’s conservative predictions in Asians, reducing the false negative rate from 22.6 to 9.7%. This was associated with a reciprocal increase in the false positive rate from 6.5 to 12.9%. Threshold adjustment was a justified solution here, as the AUROC for Asians was consistent with overall model performance (Supplementary Table 5).

AutoML performance dropped considerably in patients with an ethnicity of Other, with an AUROC of 0.79 and an F1 score of 0.52 (Supplementary Table 5). Similar results were obtained using the XGBoost model, suggesting that a reductionist label like Other is detrimental for the models. Full performance breakdowns by ethnicity are available for both models in Supplementary Tables 5 and 6.

### Analysing how our models reach their decisions

**Fig.4:**
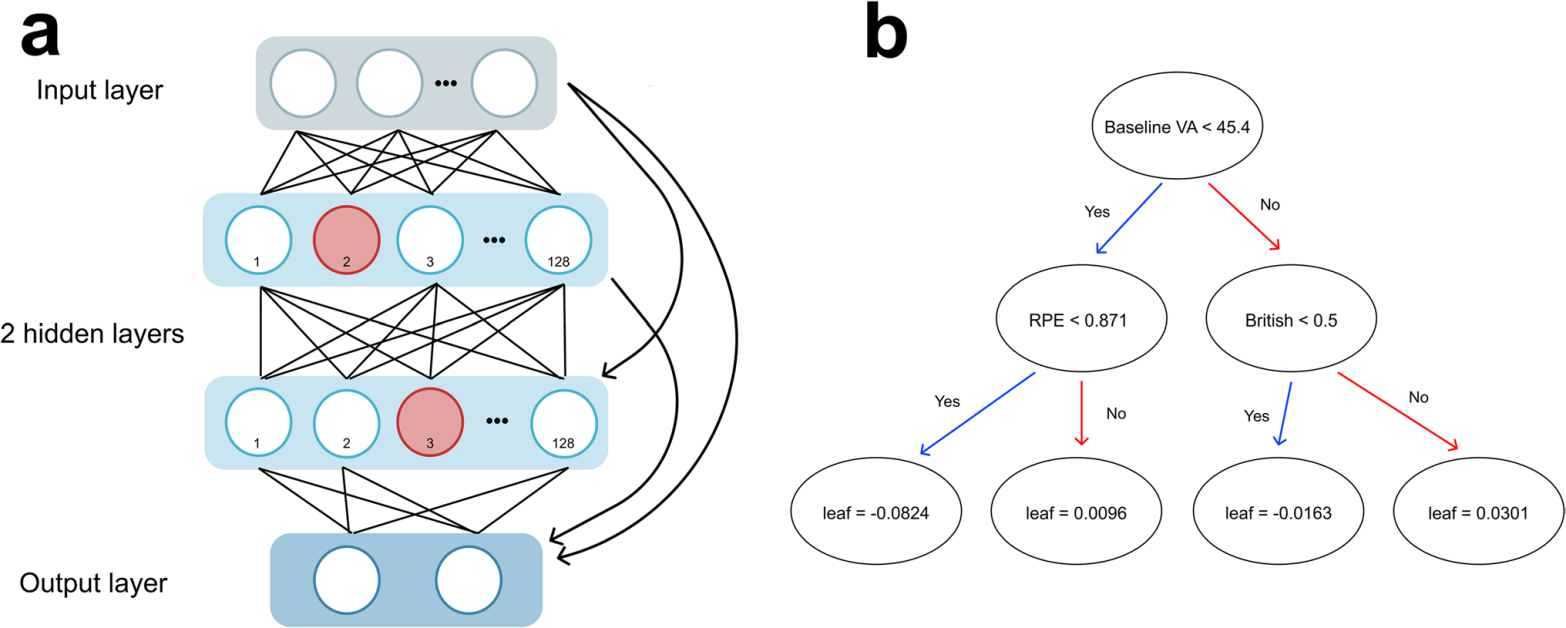
AutoML Tables and XGBoost model architectures. **a** Simplified diagram of one of the neural networks from the AutoML Tables ensemble model, consisting of one input layer, two hidden layers each with 128 nodes, a dropout of 0.25 and dense skip connections (curved arrows). Lines represent flow of information through the network from top to bottom. **b** Diagram of decision tree number 20 from the XGBoost model. Leaf values displayed are summed across all 50 trees and transformed using a logistic function to give the model’s estimated probability of an eye belonging to the ‘Above’ class. Full hyperparameter information for both models is available in Supplementary Tables 3 and 4

Analysing the Google Cloud Logs indicated that our AutoML Tables model was of an ensemble of 10 neural networks and 15 gradient-boosted decision trees. This complexity comes at the cost of interpretability, evident in the lack of information yielded by even our simplified illustration of one of the neural networks (Fig. 4a). In contrast, an advantage of XGBoost is our ability to determine how it makes its decisions by visualising a tree (Fig. 4b). However, this becomes intractable with increasingly large tree numbers.

**Fig.5:**
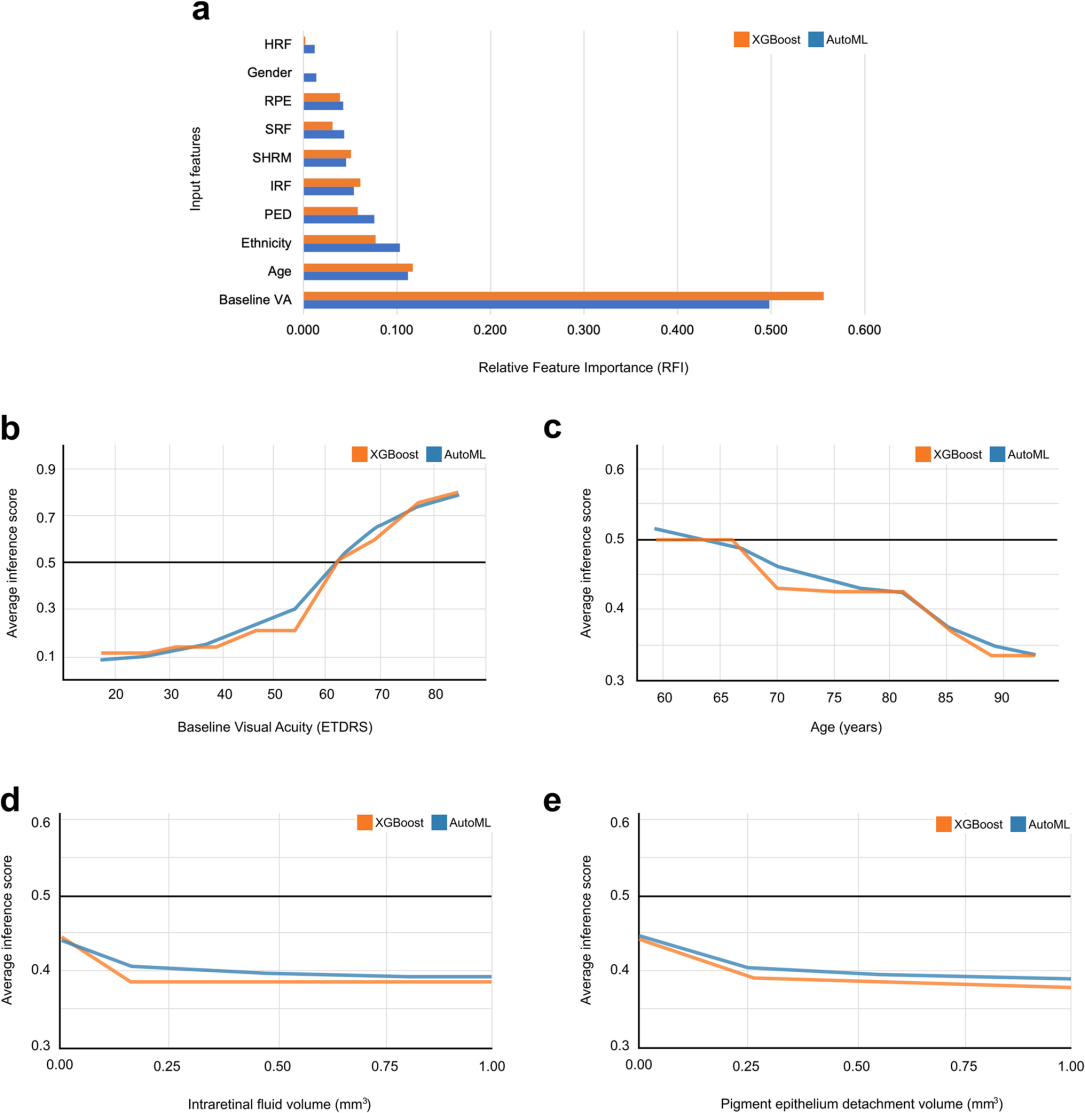
Feature importance and partial dependence plots (PDPs). **a** Relative feature importance, showing the average marginal contribution of each feature to each model’s predictions. These values were normalised to sum to 1.0 (see the ‘Methods’ section). PDPs: These show how the inference score (model’s predicted probability that an eye belongs to the ‘Above’ class) changes when a specified input feature is varied, and all other features are held at their true value. This is averaged for all datapoints in the test set to give the average inference score. The horizontal black line represents the default classification threshold of 0.5. **b** PDP for baseline VA. **c** PDP for age. **d** PDP for intraretinal fluid volume. **e** PDP for pigment epithelium detachment volume

To gain intuition for how our models reach their decisions, we analysed feature importance for both models (Fig. 5a). This highlighted baseline VA as the most important factor in determining visual outcomes, with a relative feature importance (RFI) of 0.498 and 0.556 in our AutoML and XGBoost models, respectively. This was followed by age (RFI 0.112/0.117) and ethnicity (RFI 0.103/0.077). Regarding the OCT volume measurements, AutoML Tables gave most weight to the level of PED (RFI 0.076/0.058), whilst the XGBoost model prioritised IRF levels (RFI 0.054/0.069).

As feature importance does not provide specific insight into how these features affect model predictions, we used the WIT to construct partial dependence plots (PDPs). These depict how the model’s average prediction for the test set changes as one input feature is varied and others are fixed. Crucially, this is a model agnostic technique that can be utilised to query any AutoML Tables model regardless of its architecture, as well as our XGBoost model. PDP analysis revealed a sigmoidal relationship between baseline VA and the probability of being predicted an outcome of ‘Above’ (Fig. 5b), with any increase in baseline VA between 55 and 65 being most significant. This is in line with data that suggests an average improvement in VA score of 7.2–11.3 during the first year of anti-VEGF treatment [37].

The PDP for age demonstrated that the XGBoost model’s predictions were not receptive to changes in age below 66 or between 70 and 81 years old (Fig. 5c). In contrast, any increase in age from 66 to 70 significantly decreased the probability of being classified as ‘Above’. The AutoML model shows a more consistent negative trend, likely due to the averaging effect achieved by the diverse ensemble. Notably, in both models, the probability of being predicted a positive outcome falls sharply beyond the age of 81.

The PDPs for the two most important OCT-derived measurements were highly concordant (Figs. 5d, 5e). For both IRF and PED, an initial increase from 0.0mm^3^ was associated with the models more likely to predict an outcome label of ‘Below’. However, beyond a certain point (0.15mm^3^ for IRF; 0.50mm^3^ for PED), further pathology did not affect the model’s predictions. PDPs for the remaining features are available in Supplementary Fig. 3.

### Case studies

To further elucidate the decision boundaries of our AutoML Tables model, we present case studies demonstrating where the model has classified a patient correctly or incorrectly.

**Fig.6:**
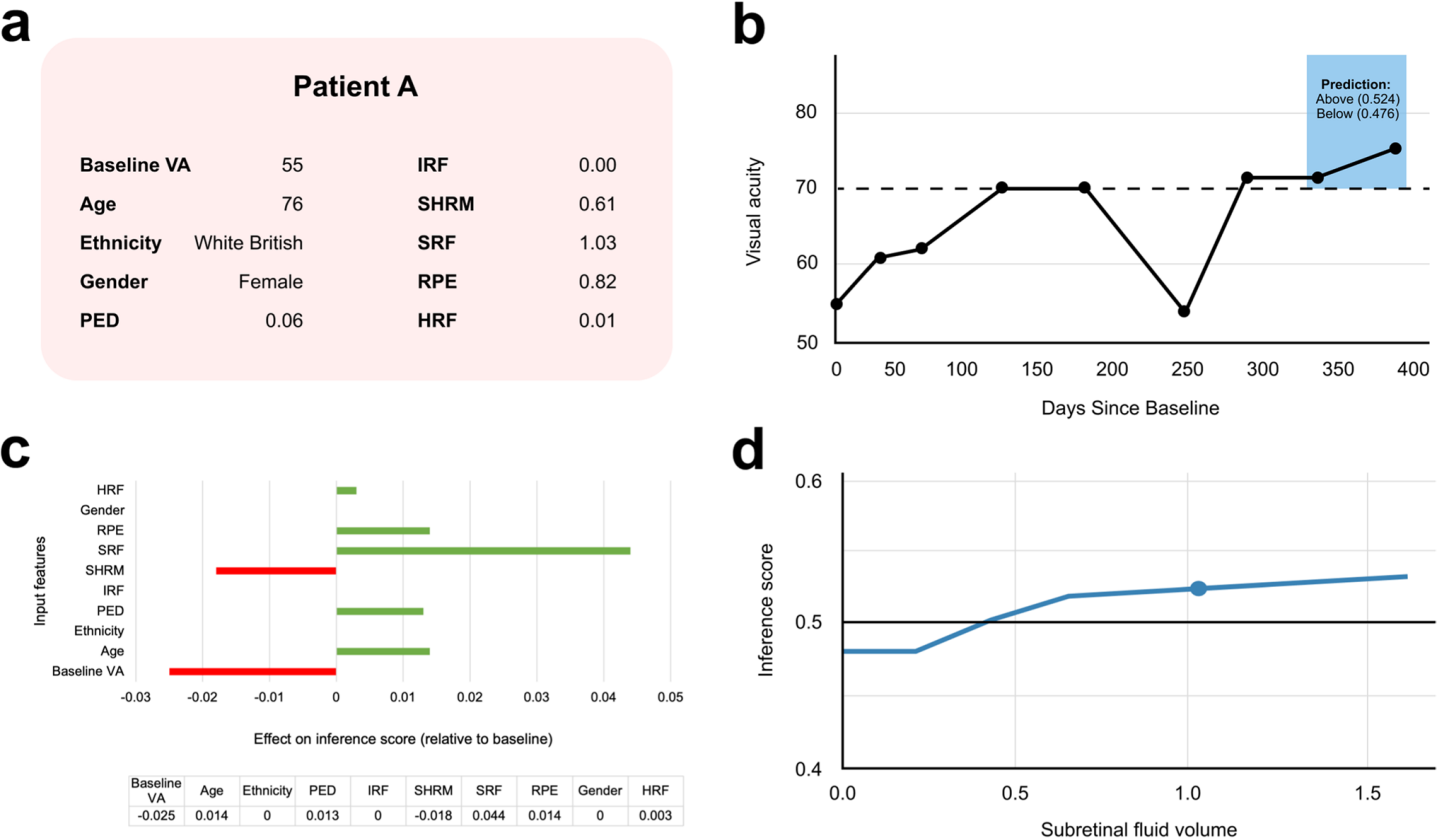
Patient A: true positive case study. **a** Input feature values for patient A. **b** VA changes throughout the first year of treatment, as measured at each follow-up appointment. NB: Only baseline information used to train model. **c** Local feature importance showing how each feature affected the AutoML model’s inference score relative to the baseline score of 0.48. **d** Individual conditional expectation (ICE) plot showing how the model’s inference score changes as SRF volume is hypothetically varied and other features are kept as shown in Fig. 6a. The indicated point represents patient A’s actual SRF volume at baseline, whilst the horizontal black line represents the default classification threshold of 0.5

Patient A is a 76-year-old White female (Fig. 6a). The patient had a baseline VA of 55—well below the threshold—but is correctly predicted to be above the threshold by 12 months (Fig. 6b). To understand why the model predicted a positive outcome, we utilised the local feature importance tool available on the AutoML Tables platform. This shows how each input feature contributed to the model’s inference score (estimated probability that an eye will be ‘Above’) of 0.524 for this patient. These contributions are determined relative to the model’s baseline inference score of 0.48, calculated using mean values for continuous input features and mode for categorical features. This demonstrated that the presence of 1.03mm^3^ of SRF was the major decision-determining factor for this patient, boosting the model’s inference score by 0.044 relative to the baseline (Fig. 6c). This is consistent with previous literature which suggests that the presence of SRF is associated with good visual outcomes [38]. We explored this further using an Individual Conditional Expectation (ICE) plot. These are similar to PDPs but for a specific patient, depicting how the model’s prediction changes when one input feature is varied whilst others are fixed at their true values. This revealed a decision boundary at 0.4mm^3^ SRF (Fig. 6d).

**Fig.7:**
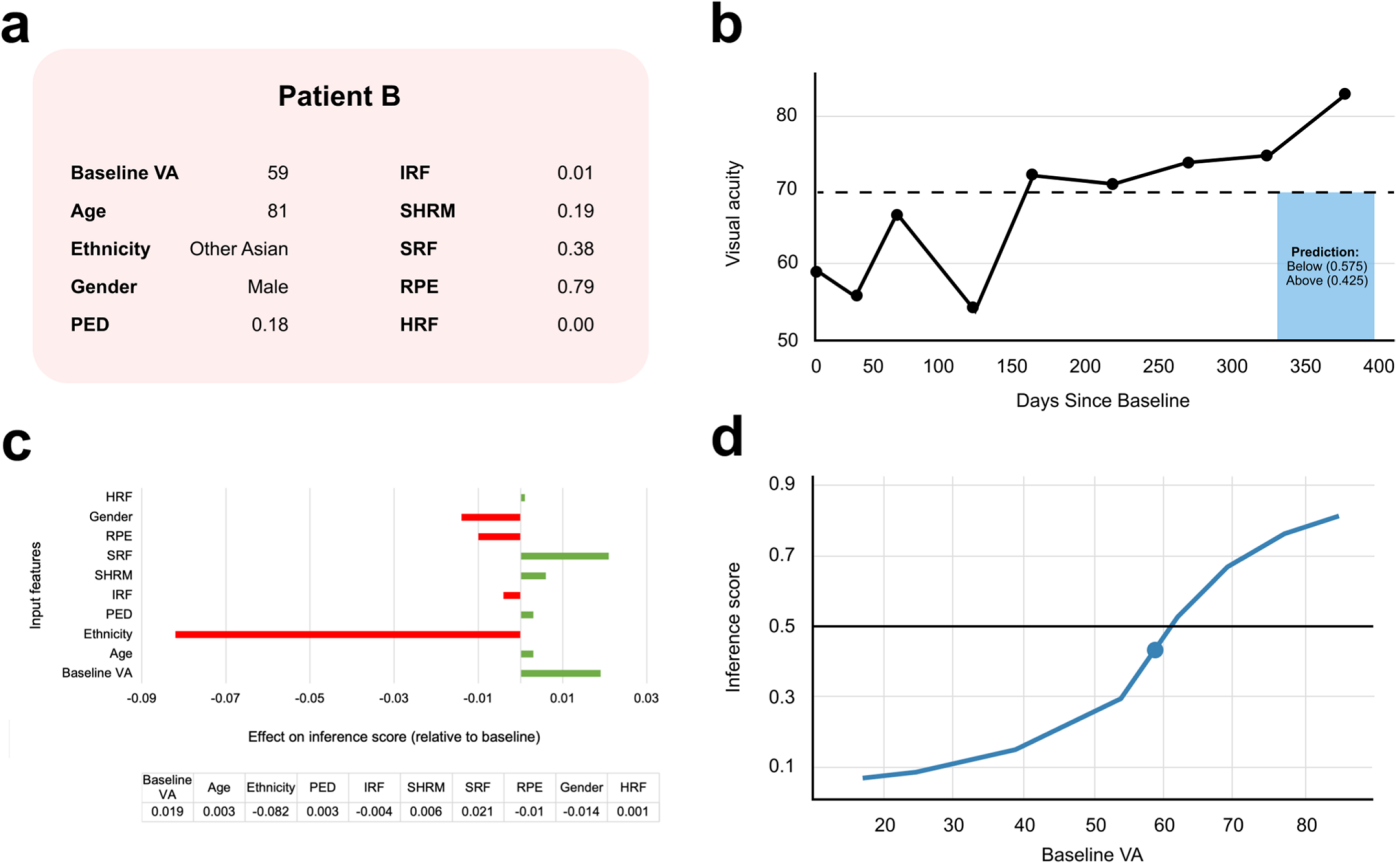
Patient B: false negative case study. **a** Input feature values for patient B. **b** VA changes throughout first year of treatment. **c** Local feature importance. **d** ICE plot for baseline VA. The indicated point represents patient B’s actual baseline VA

We also present patient B, an 81-year-old Asian male (Fig. 7a). Local feature importance highlights the patient’s ethnicity as the critical factor in the model wrongly predicting an outcome of ‘Below’ (Fig. 7c). Using the WIT, we hypothetically changed his ethnicity to British and found the model now predicted an outcome of ‘Above’. Moreover, this patient’s ICE plot for baseline VA demonstrates that a very small change in this value would have flipped the model’s prediction (Fig. 7d). This is important considering the small element of human error associated with the measurement of VA [39].

## Discussion

In this study, we have evaluated an AutoML Tables model for the binary classification task of predicting whether patients with nAMD will have a visual acuity above or below a threshold VA score of 70, one year after initiating anti-VEGF treatment.

We have demonstrated that an AutoML Tables model can achieve similar results to a manually designed bespoke algorithm without necessitating coding expertise. Our model also significantly improves upon a previously reported model predicting similar outcome measures with an AUROC of 0.78 [9]. Our work represents a framework whereby clinical researchers with limited coding skills may utilise ML techniques to test their own hypotheses.

Patients newly diagnosed with nAMD are typically confronted with a high degree of uncertainty regarding their prognosis [8]. Our model helps address this problem, demonstrating the potential for instant and personalised vision predictions at initiation of treatment. A prediction of ‘Above’ could promote treatment adherence and provide reassurance to patients regarding their eyesight, which is known to be positively associated with quality of life [40]. Our model’s high specificity is also desirable for patients with worse predicted visual outcomes, as early mental and occupational health support is beneficial to those patients [41].

Thus far, the implementation of ML in healthcare settings remains uncommon [42]. Barriers to clinical implementation include the lack of insight into how models reach their decisions and concerns regarding hidden stratification as a source of bias [43]. This motivated us to establish an AutoML-WIT pipeline (video available in Supplementary Information) that facilitates model interpretability.

Using the WIT, we found that predictions were pessimistic for Asian patients and that models performed worse in the Other ethnic group. More than 96% of participants in the major MARINA and ANCHOR anti-VEGF clinical trials were of White background [44]. Thus, clinical implementation of this model without first addressing these issues risks further reinforcing the disparities of non-White patients with regard to anti-VEGF treatment. Threshold adjustment using the WIT was able to rectify the high false negative rate in Asian patients. In contrast, the poor performance in the Other ethnic category represents a constraint by the available retrospective ethnicity data, which follows the UK Census system [45].

As expected, baseline VA was the most important input feature for our models. This was followed by age and ethnicity, a possible indication as to why our model exceeded the performance of a previously reported model that did not include demographic data [9]. The importance of ethnicity to our models merits further enquiry, as recently a systematic review of 30 studies looking into factors that affect VA outcomes in nAMD found that none had studied the effect of ethnicity [10]. The OCT-derived measurements played a small but significant role in model predictions, with a notable exception in the case of HRF, which was of negligible importance.

Through local feature importance and ICE plots, we further show that our AutoML-WIT pipeline is able to provide comprehensive insight into why individual patients were predicted to be above or below the VA threshold. Our false negative case study raises the question of how this information would be handled in clinical practice. Given the detrimental effect that patient B’s ethnicity had on the model’s prediction, how will doctors determine when to overrule model predictions that seem irrational?

## Limitations

Our study has several limitations. We excluded patients that lacked a follow-up appointment at one year. This may introduce a selection bias, as it likely has a disproportionate effect on patients with poor visual outcomes who find it more difficult to attend appointments. Our sample size of 1631 is relatively small for training an ML task, and better performance is likely to be dependent on securing larger, national datasets. A small sample size limits the scope to analyse performance by ethnic group; for example, there were only 15 eyes belonging to Black patients present in our test set. This also increases the likelihood of imbalances, such as the higher proportion of Asian patients with good VA outcomes in our test set compared to the training set. Moreover, results from a model trained on MEH patients—though clinically representative of the nAMD demographic—may not generalise well to other care settings which adopt different treatment protocols.

As we utilised real-world data, variation in treatment protocols was observed amongst MEH patients. One strategy to mitigate this may be adjusting for additional confounding variables, such as injection frequency. However, our primary focus was a comparative analysis of AutoML and how the WIT facilitates the investigation of algorithmic bias, rather than to build the most robust and generalisable model possible.

Finally, it is important to clarify that, whilst our AutoML model was able to achieve an AUC of 0.849, such models are not yet ready to incorporate into clinical practice. The sensitivity of our model was 0.69, suggesting a notable risk of false negative predictions. Nevertheless, the future potential for AutoML adoption in clinical practice is optimistic, considering the rapid rate at which these platforms are improving and the increasing availability of larger datasets.

## Conclusion

In summary, our work builds upon the growing body of literature regarding AutoML in healthcare. The majority of this research has focused on image data. Here, we demonstrate an interpretable AutoML Tables model which can predict VA outcomes in patients with nAMD using a structured dataset. This type of data exists in abundance in electronic health records. Thus, there is significant opportunity for future work to utilise our AutoML-WIT pipeline to develop high performing, interpretable ML models with minimal coding.

## Supplementary Information

Below is the link to the electronic supplementary material.

Supplementary file1 (DOCX 678 KB)

Supplementary file2 (M4V 50965 KB)

## Data Availability

The data used in this study is based on the same patient cohort as Fu et al. [9]. De-identified data from that study has been made publicly available on the Dryad Digital Repository: https://doi.org/10.5061/dryad.573n5tb5d.

## Code availability

Code from this study is available at https://github.com/abdallahabbas20/nAMD_AutoML_Study_Code.

## Declarations

### Disclaimer

 Dr Keane has acted as a consultant for DeepMind, Roche, Novartis, Apellis, and BitFount and is an equity owner in Big Picture Medical. He has received speaker fees from Heidelberg Engineering, Topcon, Allergan, and Bayer. Dr Keane is supported by a Moorfields Eye Charity Career Development Award (R190028A) and a UK Research & Innovation Future Leaders Fellowship (MR/T019050/1).Dr Fu is supported by Moorfields Eye Hospital and has acted as a consultant for Abbvie, Allergan, DeepMind. Dr. Korot has acted as a consultant for Genentech, Alimera, and Google Health, and is an equity owner in Reti Health. He was previously supported by the Moorfields Eye Charity Springboard Grant R190016A. All other authors have no disclosures.
