# Supplementary material for: Evaluating an automated machine learning model that predicts visual acuity outcomes in patients with neovascular age-related macular degeneration
Source: Graefes Arch Clin Exp Ophthalmol. 2022 Feb 5;260(8):2461–73. doi: 10.1007/s00417-021-05544-y (PMC9325856; doi:10.1007/s00417-021-05544-y)
Supplement: Supplementary file 1 — Supplementary file1 (DOCX 678 KB) [file 417_2021_5544_MOESM1_ESM.docx]

# **Supplementary material**

**Journal:** Graefe's Archive for Clinical and Experimental Ophthalmology

**Title:** Evaluating an automated machine learning model that predicts visual acuity outcomes in patients receiving treatment for neovascular age-related macular degeneration

**Authors:** Abdallah Abbas, Ciara O’Byrne, Dun Jack Fu, Gabriella Moraes, Konstantinos Balaskas, Robbert Struyven, Sara Beqiri, Siegfried K. Wagner, Edward Korot, Pearse A. Keane

**Corresponding author:**

Abdallah Abbas

Institution: University College London Medical School, London, UK

Email: Abdallah.abbas.18@ucl.ac.uk


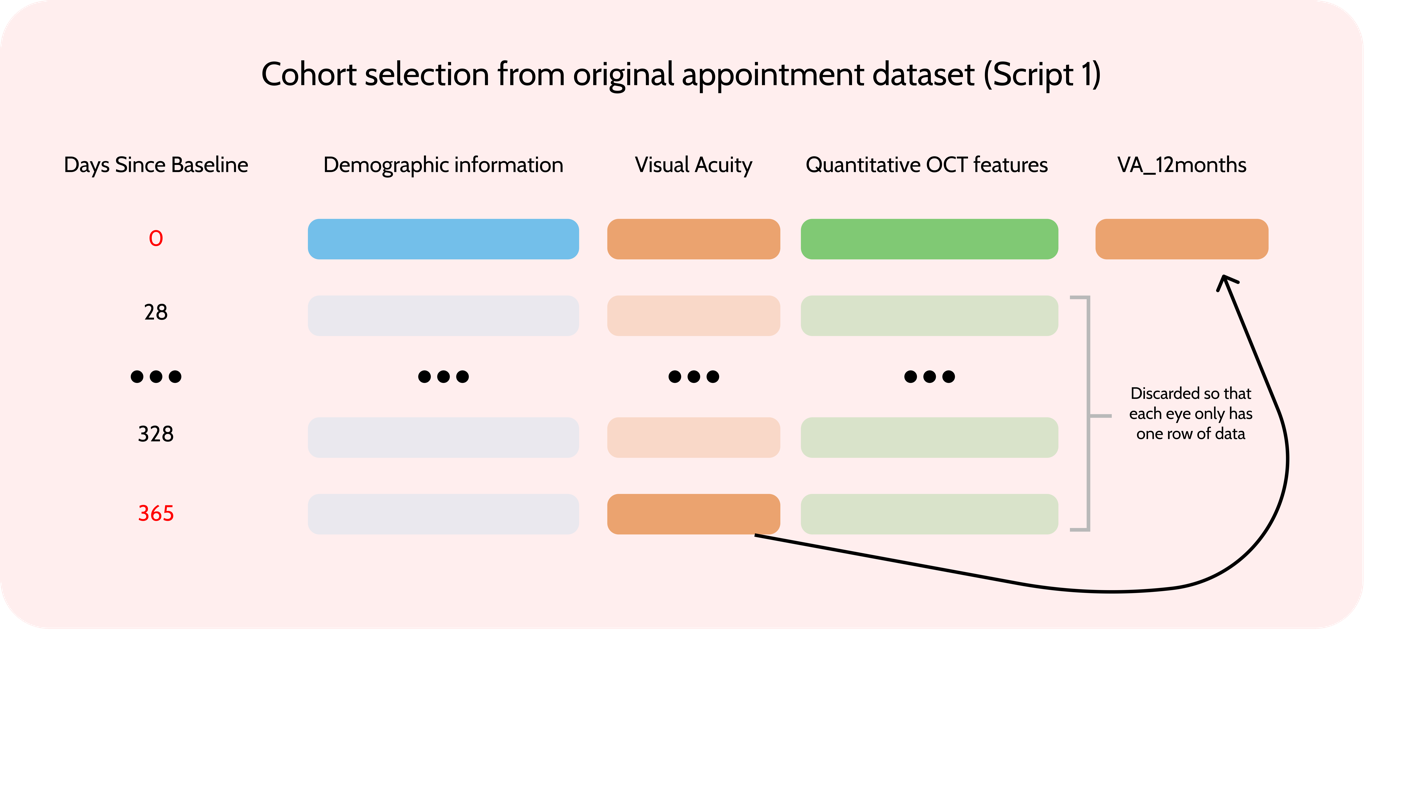


**Supplementary Fig.1** Cohort selection process. We built a program to sort through the appointment data and select appointments corresponding to patients receiving their first anti-VEGF injection (where “DaysSinceBaseline” = 0). For each of these, the program searches for a follow-up appointment for the same eye one year later and appends the VA score from this follow-up to the baseline data. As appointment schedules vary, any follow-up appointment between 335 and 395 days from baseline was considered. If a patient attended multiple appointments within this window the visit closest to 365 days was selected. Whilst the dataset contained information about the fellow eye (which often converts to AMD), to standardise our sample we only selected the first-treated eye, unless both eyes were treated simultaneously. NB: This figure shows the process for one patient

**
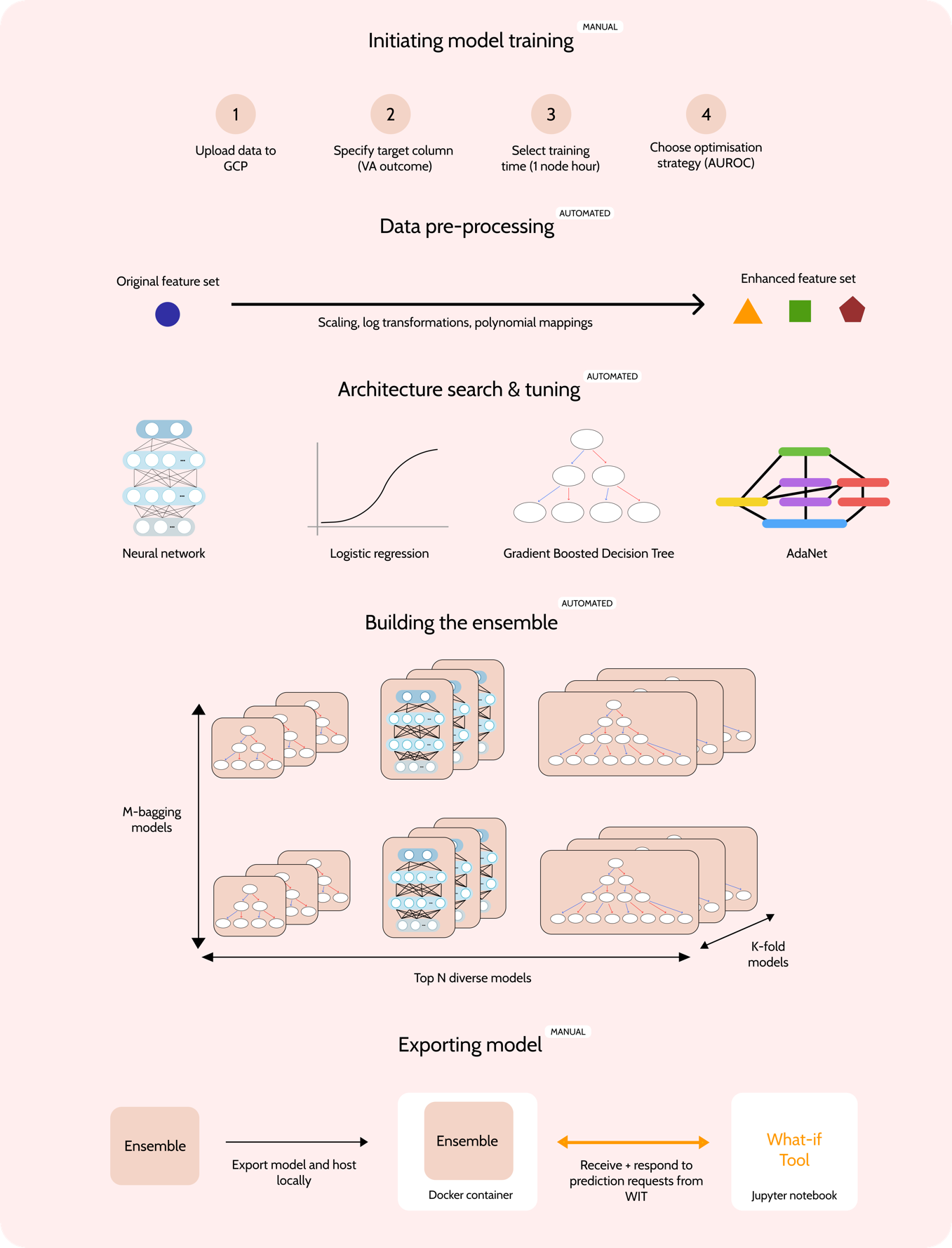
**

**Supplementary Fig.2** The AutoML Tables pipeline. Initiating model training: We uploaded our training data to the GCP and selected our target column, training time and optimisation objective. *Data pre-processing:* Common data transformations (e.g., log values/z-scores) are taken of the feature columns to generate an enhanced feature set. *Architecture search and tuning:* A range of model architectures are scanned and trialled by a ‘Controller’ network. Hyperparameter tuning is also carried by adjusting model-specific parameters such as tree depth or hidden layer size for the different trials. The performance of each trial is evaluated and fed back to the controller network as a ‘reward’ signal. *Building the ensemble:* The N best performing models are selected, cross-validated using K-folds and bagging* with M samples to create a diverse ensemble model that takes the average of the K x N x M outputs. *Exporting model:* We exported the trained model and served it within a Docker container using the Command Line Interface (CLI), ready to receive prediction requests for WIT analysis. *Bagging (bootstrap aggregating) involves sampling with replacements to train multiple models on M slightly different variations of the training data


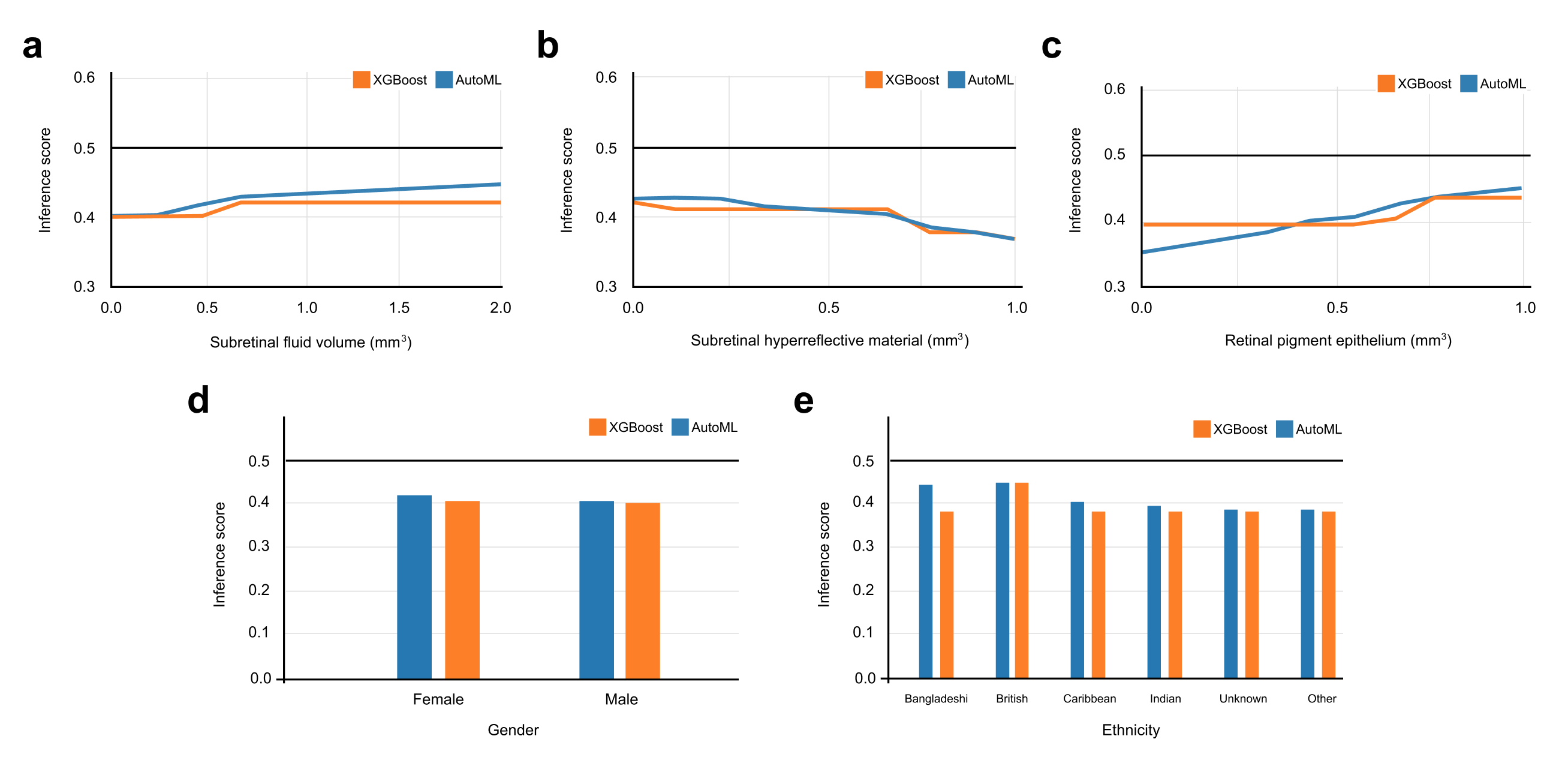


**Supplementary Fig.3** PDPs for remaining input features. **a)** PDP for retinal pigment epithelium (mm^3^). **b)** PDP for subretinal hyperreflective material (mm^3^). **c)** PDP for subretinal fluid (mm^3^). **d)** PDP for gender. **e)** PDP for ethnicity. Here we are limited by the WIT which only displays six ethnicities as part of the graph

| OCT Feature | Acronym | Description |
| --- | --- | --- |
| Intra-retinal fluid | IRF | Fluid that builds up inside the retinal layers, appearing as areas of oval or round hypo-reflectivity within the neurosensory retina. |
| Subretinal fluid | SRF | Hypo-reflective areas of serous fluid build-up in the subretinal space (i.e., between the neurosensory retina and the retinal pigment epithelium). |
| Subretinal hyperreflective material | SHRM | Areas of hyperreflectivity between the retina and retinal pigment epithelium (this can constitute a range of materials e.g., fibrosis or neovascular material). |
| Hyperreflective foci | HRF | Small, distinct lesions within the retina that are hyperreflective. |
| Retinal pigment epithelium | RPE | A pigmented layer of cells underlying the neurosensory retina, appears as a hyperreflective band. |
| Pigment epithelium detachment | PED | Elevation of the retinal pigment epithelium (normally dome shaped but could be irregular) relative to Bruch’s membrane. |

**Supplementary Table 1.** Descriptions of the OCT-derived features selected as input features for model training

|  | | |  | | Total (n=1631) | Train (n=1386) | Test (n=245) |
| --- | --- | --- | --- | --- | --- | --- | --- |
| Age (years) | | |  | |  |  |  |
|  |  | | | | 80 (73-85) | 80 (73-85) | 80 (73-84) |
| Ethnicity | | |  | |  |  |  |
|  | | **White**  White British  White Irish  **Asian**  Indian  Chinese  Pakistani  Bangladeshi  Other Asian  **Black**  Caribbean  African  **Other**  **Unknown** | | | **868 (53.2%)**  813 (49.8%)  55 (3.4%)  **170 (10.4%)**  99 (6.1%)  12 (0.7%)  12 (0.7%)  10 (0.6%)  37 (2.2%)  **33 (2.0%)**  17 (1.0%)  16 (1.0%)  **380 (23.3%)**  **180 (11.0%)** | **734 (53.0%)**  686 (49.5%)  48 (3.5%)  **139 (10.0%)**  88 (6.3%)  10 (0.7%)  9 (0.6%)  6 (0.4%)  26 (1.9%)  **28 (2.0%)**  14 (1.0%)  14 (1.0%)  **330 (23.8%)**  **155 (11.1%)** | **134 (54.7%)**  127 (51.8%)  7 (2.9%)  **31 (12.7%)**  11 (4.5%)  2 (0.8%)  3 (1.2%)  4 (1.6%)  11 (4.5%)  **5 (2.0%)**  3 (1.2%)  2 (0.8%)  **50 (20.4%)**  **25 (10.2%)** |
| Gender | | |  | |  |  |  |
|  | | Female  Male | | | 988 (60.6%)  643 (39.4%) | 849 (61.3%)  537 (32.9%) | 139 (56.7%)  106 (43.3%) |
| Baseline VA (ETDRS) | | | |  |  |  |  |
|  | |  | | | 58 (46-68) | 58 (46-68) | 56 (46-68) |
| OCT features (mm^3^) | | | |  |  |  |  |
|  | | RPE  IRF  SRF  HRF*  SHRM  PED | | | 0.81 (0.77-0.86)  0.00 (0.00-0.08)  0.20 (0.03-0.57)  0.00 (0.00-0.00)  0.13 (0.02-0.41)  0.37 (0.13-0.90) | 0.81 (0.77-0.86)  0.00 (0.00-0.08)  0.20 (0.03-0.57)  0.00 (0.00-0.00)  0.14 (0.02-0.42)  0.37 (0.13-0.88) | 0.81 (0.77-0.86)  0.01 (0.00-0.09)  0.21 (0.02-0.57)  0.00 (0.00-0.00)  0.12 (0.01-0.35)  0.40 (0.14-0.96) |

**Supplementary Table 2.** VA outcome and input feature summary statistics categorised by train and test splits. Continuous features presented as median (IQR). Models were trained using ethnicities in accordance with definitions outlined by the UK Census and the NHS Data Dictionary. For analysis, these were grouped into White, Black, Asian, Other or Unknown (shown in bold) in line with the groupings also stated by the UK Census system. *HRF to 5.d.p (Total = 0.00071 (0.00021- 0.00236); Train = 0.00073 (0.00022 - 0.00236), and Test = 0.00063 (0.00018 - 0.00237)

| **Neural network hyperparameters** | **0** | **1** | **2** | **3** | **4** | **5** | **6** |
| --- | --- | --- | --- | --- | --- | --- | --- |
| Hidden layer size | 128 | 128 | 64 | 64 | 128 | 64 | 16 |
| Number of hidden layers | 2 | 2 | 2 | 2 | 2 | 2 | 1 |
| Dropout rate | 0.25 | 0.25 | 0.125 | 0.125 | 0.25 | 0.125 | 0 |
| Skip connections type | dense | dense | concat | concat | dense | dense | concat |
| Number of cross layers | 0 | 0 | 2 | 2 | 0 | 2 | 2 |

| **NN hyperparameters** | **7** | **8** | **9** | **10** | **11** | **12** | **13** | **19** |
| --- | --- | --- | --- | --- | --- | --- | --- | --- |
| Hidden layer size | 128 | 64 | 16 | 16 | 16 | 64 | 16 | 64 |
| Number of hidden layers | 2 | 2 | 1 | 1 | 1 | 2 | 1 | 2 |
| Dropout rate | 0.25 | 0.125 | 0 | 0 | 0 | 0.125 | 0 | 0.25 |
| Skip connections type | dense | concat | concat | concat | concat | concat | concat | dense |
| Number of cross layers | 0 | 2 | 2 | 2 | 2 | 2 | 2 | 0 |

| **Gradient Boosted Decision Tree**  **Hyperparameters** | **Models 14 - 18** | **Models 20 - 24** |
| --- | --- | --- |
| Number of trees | 250 | 400 |
| Max tree depth | 3 | 6 |
| Tree L1 Regularisation | 3 | 3 |
| Tree L2 Regularisation | 3 | 3 |
| Centre Bias | False | False |

**Supplementary Table 3.** Final hyperparameters for the AutoML Tables model. These were obtained by analysing the Google Cloud Logs and show that the model was an ensemble composed of 15 neural networks and 10 gradient boosted decision trees

| **Hyperparameters for XGBoost model** | | | |
| --- | --- | --- | --- |
| N_estimators | 50 | Colsample_by_tree | 0.8 |
| Eta | 0.1 | Subsample | 1.0 |
| Max_depth | 2 | Min_child_weight | 3.5 |
| Gamma | 0.8 | Objective | Binary:logistic |

**Supplementary Table 4.** Final hyperparameters for XGBoost model. Obtained via a grid search with stratified cross validation (Script 2). Descriptions of hyperparameters are available at: https://xgboost.readthedocs.io/en/latest/parameter.html

| AutoML | AUROC | Sensitivity | Specificity | PPV | NPV | Accuracy | F1 score |
| --- | --- | --- | --- | --- | --- | --- | --- |
| White (n=134) | 0.86 | 78.6% | 78.2% | 72.1% | 83.6% | 78.4% | 0.75 |
| Asian (n=31) | 0.86 | 56.3% | 86.7% | 81.8% | 65% | 71.1% | 0.67 |
| Black (n=5) | 1.00 | 100% | 100% | 100% | 100% | 100% | 1.00 |
| Other (n=50) | 0.79 | 46.8% | 85.7% | 58.3% | 78.9% | 74.0% | 0.52 |
| Unknown (n=25) | 0.86 | 60.0% | 86.7% | 75.0% | 76.5% | 76.0% | 0.67 |

| XGBoost | AUROC | Sensitivity | Specificity | PPV | NPV | Accuracy | F1 score |
| --- | --- | --- | --- | --- | --- | --- | --- |
| White (n=134) | 0.86 | 76.8% | 79.5% | 72.9% | 82.7% | 78.4% | 0.75 |
| Asian (n=31) | 0.86 | 56.3% | 100% | 100% | 68.1% | 77.4% | 0.72 |
| Black (n=5) | 1.00 | 100% | 100% | 100% | 100% | 100% | 1.00 |
| Other (n=50) | 0.79 | 46.8% | 85.7% | 58.3% | 78.9% | 74.0% | 0.52 |
| Unknown (n=25) | 0.85 | 50.0% | 93.3% | 83.3% | 73.7% | 76.0% | 0.63 |

**Supplementary Tables 5 & 6.** Performance metrics for AutoML Tables and XGBoost models broken down by ethnic group
